# Effectiveness of clinical scenario dramas to teach doctor-patient relationship and communication skills

**DOI:** 10.1186/s12909-020-02387-9

**Published:** 2020-11-26

**Authors:** Yinan Jiang, Lili Shi, Jinya Cao, Liming Zhu, Yue Sha, Tao Li, Xiaohong Ning, Xia Hong, Xiaoyan Dai, Jing Wei

**Affiliations:** 1 Department of Psychological Medicine, Peking Union Medical College Hospital, Chinese Academy of Medical Sciences & Peking Union Medical College, Shuaifuyuan1, Dongcheng District, Beijing, 100730 PR China; 2 Department of Gastroenterology, Peking Union Medical College Hospital, Chinese Academy of Medical Sciences & Peking Union Medical College, Shuaifuyuan1, Dongcheng District, Beijing, 100730 PR China; 3 Department of General Internal Medicine, Peking Union Medical College Hospital, Chinese Academy of Medical Sciences & Peking Union Medical College, Shuaifuyuan1, Dongcheng District, Beijing, 100730 PR China; 4 Department of Medical Oncology, Peking Union Medical College Hospital, Chinese Academy of Medical Sciences & Peking Union Medical College, Dongcheng District, Beijing, 100730 PR China; 5 International Medical Services, Peking Union Medical College Hospital, Chinese Academy of Medical Sciences & Peking Union Medical College, Dongcheng District, Beijing, 100730 PR China

**Keywords:** Doctor-patient relationship, Clinical scenario drama, Communication skills

## Abstract

**Background:**

The doctor-patient relationship in China has deteriorated in recent years, and poor doctor-patient communication is one of the main reasons. How to effectively carry out doctor-patient communication training originated from the West among Chinese medical students still to be studied. In the past decade, Peking Union Medical College has adopted clinical scenario drama to teach doctor-patient relationship and clinical communication skills. The aim of this study was to introduce clinical scenario dramas and evaluate its effectiveness in promoting doctor-patient relationships and clinical communication skills through students’ self-perceptions in Chinese medical students.

**Methods:**

This study was a retrospective, self-controlled study and conducted from March 2009 to October 2018. Doctor-patient relationship and communication skills training were administered to all sixth-year medical students, which involved lectures and various clinical scenario dramas. The program totaled 24 h, of which each class session was 3 h, with 8 sessions in total. All students were requested to complete an anonymous 5 likert self-rating survey including self-confidence in using communication skills and self-perceived learning attitude and ability before and at the end of the course. In addition, they were requested to evaluate the curriculum after completion of the course.

**Results:**

Clinical scenario dramas helped students improve their self-confidence in clinical communication skills except for psychosomatic history taking (*p* < 0.05). The interests for participation in clinical scenario dramas were higher compared to attending lectures (4.39 ± 0.610 Vs 4.07 ± 0.831, *p*<0.01). Study participants were highly satisfied in the course setting, teaching instructors and content (4.61 ± 0.546, 4.65 ± 0.535, 4.63 ± 0.534). The self-evaluation results demonstrated that clinical scenario dramas improved the learning ability of medical students (*p* < 0.05).

**Conclusion:**

The use of clinical scenario dramas was helpful in teaching doctor-patient communication skills.

**Supplementary Information:**

**Supplementary information** accompanies this paper at 10.1186/s12909-020-02387-9.

## Background

In recent years, doctor-patient relations in China are deteriorating [1–4]. Doctors in China often face threats of violence and patients distrust the current health care system. According to a 2014 study of community hospitals in Heilongjiang Province, that 42.2% of medical personnel experienced violent attacks in the past year [5]. And researches indicate that doctor-patient disputes in China are largely due to poor doctor-patient communication [6, 7]. Both medical staff [8] and patients [6, 9] feel that there are serious problems in doctor-patient communication.

Previous studies from the West have shown that doctor-patient communication is influenced by many factors, including physician and patient communication style, patients’ demographic characteristic, and demographic concordance between physician and patient [10–13]. But what are the barriers to good communication between Chinese doctors and their patients? In ancient China, the position of doctors was theocratic and patriarchal [14], so there was absolute dominance between doctors and patients. However, with the economic development in recent years, Chinese patients have a stronger sense of autonomy and rights protection. Similarly, previous studies in other Asian countries have found that under the collectivist view, patients are conceptualized as subordinates of doctors, who are more authoritative and dominate the discourse in medical consultations [15–17].

As we all know, good doctor-patient communication has the potential to help ease patient’s emotions, facilitate patient’s comprehension of medical information and improve in identifying the patients’ needs, perceptions, and expectations [18–21]. But doctor-patient communication skills are not innate. Traditionally, Chinese doctors’ communication ability can only be achieved through self-exploration in clinical practice. In the past decade, doctor-patient communication training courses [22, 23] have been introduced to China from western countries. However, due to the differences between Eastern and Western cultures, whether these courses can be well accepted by Chinese medical students remains an open question. In addition, compared with the West, the Chinese people have a different understanding of education. In 《Origin of Chinese Characters (Shuo wen jie zi)》, Education (Jiao Yu) is defined as: Teaching (Jiao) is that students are to behave by following dictations from teachers; fostering (Yu) aims at leading children to be a man of virtues. Previous studies have shown that in China, doctor-patient communication training programs are limited in both medical school and graduate education, and they emphasize more on theoretical knowledge than practical training of communication skills [24]. Communication training not only need technical training, but more importantly helps students to explore the inner experience of people in different roles.

Based on the above reasons, beginning in 2007, the department of psychological medicine at the Peking Union Medical College Hospital (PUMCH) began teaching courses in doctor-patient communication and using clinical scenario dramas as a teaching method. Clinical scenario drama is rooted in roles theory, and it explores the experiential process of conflict and transformation of doctor-patient relationship with the help of dramatic technology. Its advantage is that it can help medical students who have insufficient experience of doctor-patient relationship to better understand the inner experience of different roles in doctor-patient relationship [25]. This study aimed to introduce clinical scenario drama and to evaluate the acceptability and usefulness of the program through medical students’ self-perceptions.

## Methods

The study protocol was approved by the Ethics Committees of the Peking Union Medical College Hospital, Beijing.

### Study design

The study was a pre-post intervention study and conducted from March 2009 to October 2018.

### Inclusion and exclusion criteria

Inclusion criteria were as follows: all sixth-year medical students from Peking Union Medical College (equivalent to third-year medical students in American medical colleges) participated in the medical clinical communication skills course.

Exclusion criteria: students who did not attend classes due to illness, international exchange programs, etc.

### Course setting

The clinical communication skills course was a compulsory course in PUMC. The proposal to conduct the course was reviewed and accepted by the education committee of PUMCH. The clinical communication skills course included a total of 24 h of teaching, of which each class session was 3 h, with a total of 8 sessions. According to Calgary-Cambridge Guide and the actual situation of clinical practice in China [26–28], seven topics were covered in the curriculum, i.e., relationship building and empathy, obtaining patient psychosomatic history, explaining medical problems, negotiating treatment options, communicating bad news, coping with demanding patients, and communicating with relatives. Teaching consisted of 30-min of lecture and 2.5-h of clinical scenario drama for each session. Fifty to one hundred students for each grade were enrolled in the course and were divided into 2–4 groups.

### Teaching instructors

Every group was instructed by two trained doctors who were experienced in conducting clinical scenario drama courses, small-group teaching methods and communication techniques. One of the teachers was a psychosomatic medical doctor who was very experienced in handling difficult conversations with patients and families and had over 10 years of experience. The other teacher was an internal medicine doctor from the PUMC department that specialized in gastrointestinal, general practice, oncology, geriatric or palliative medicine. All instructors had participated in an advanced communication training program that was funded by the Chinese Medical Doctor Association (CMDA). The program was a two 5-day retreat that focused on instructing clinical doctors how to teach communication skills to physicians-in-training.

### Clinical scenario dramas

Below we describe the elements of a typical clinical scenario drama.

### Script

There were three standardized scripts for each topic (Table 1), with two scripts being used during a 2.5-h clinical scenario drama. The content emphasized on doctor-patient communication in clinical practice. The scripts entailed realistic situations faced by doctors and their patients. Students were required to use the communication skills learned from the course to resolve difficulties and miscommunications typically faced by doctors and patients.

**Table 1:** Standardized scripts for each topic

Background of communication	Script conflict	Communications model
Fever of unknown origin	In the face of medical uncertainty	Relationship building and empathy
The fever after visiting a farm	The patient’s dilemma: His/her own medical history may affect his/her uncle’s farm	Obtaining patient’s psychosomatic history
A gallstone patient whose father died on the operation table	How to identify the fear inside the patient	Explaining medical problems & Negotiating treatment options
A woman with advanced ovarian cancer expects to attend her daughter’s wedding next year	How to deliver bad news	Breaking bad news
The professor went out for a meeting without informing the patient, and the resident doctor received the patient	Dealing with disappointed and angry patients	Coping with demanding patients: CALM model
The patient was diabetic but refused to take medication. The daughter wanted the doctor to order the patient to take medication	How can doctor establish a good doctor-patient relationship with an unwilling patient	Communicating with family

### Rehearsal

Group members volunteered or were invited to play characters in the scenarios. Volunteers were helped by instructors to understand their roles with respect to the illness studied; the background of the patient’s character, including age, gender, occupation, and demeanor; as well as the details regarding the patient’s illness and enact their condition (Table 2). Volunteers had to place themselves in the shoes of the patient to understand the communication challenges they would experience as they describe their medical situation, emotional state, and key relationships. Non-character group members had to observe the rehearsal and design the stage background, light and acoustics, and so on.

**Table 2:** Standard operating procedure for role coaching

Role coaching for ‘doctors’	Role coaching for ‘patients’
1. The ‘doctor’ reads the script independently 2. Ask the ‘doctor’ to give a brief description of the information obtained in the first-person narration 3. If necessary, help the ‘doctor’ review the relevant medical knowledge and the key points of communication skills. 4. Inform learning tasks a) In the following exercise, your task is … b) Your feedback is important for learning, and try to remember the successes and difficulties in completing the task c) Notice: In role coaching, the patient will be asked not to embarrass the ‘doctor’ in the medical framework, but if your communication style does not satisfy him, he can express it in his own way 5. Help the ‘doctor’ get into character: “Dr. X, you are about to meet the patient. How are you feeling?”	1. The ‘patient’ reads the script independently 2. Ask the ‘patient’ to give a brief description of the information obtained in the first-person narration 3. According to the script, propose a hypothetical scenario “How would you react if the doctor said … “, promote students to deeply understand the feelings of the role. Similar medical or life experiences may help ‘patient’ get into the role. 4. Get out of the script: The script is just a frame of reference, you are the protagonist in the drill, you can make response according to your own personality and feelings at that time.” 5. Inform learning tasks: a) Your feedback is important to learning. Try to keep in mind what makes you feel good or bad while the doctor is doing the job. b) Don’t embarrass the doctor within the medical framework, but if his or her performance doesn’t satisfy you, you can express it in your own way. 6. Ask about feelings of the ‘patient’: “Mr. / Mrs. X, you are going to see the doctor. How are you feeling?”

### Clinical scenario dramas

The stage directions for clinical scenario dramas were quite flexible. For example, the scene could be performed on a riser in front of an audience or in a circle surrounded by an audience. Depending on the scenario, the houselights were slightly dimmed or the room immersed in total darkness with spotlights to illuminate the stage. Furthermore, music could be used in the background, chairs and other props could be used, and group members could create imaginary settings, e.g., a hospital room, clinic, or office for the scenario. If needed, a brief introductory statement that sets the scene could be provided; for example, “In this scene, we will meet Mr. Lee, a middle-aged male patient who is angry because his doctor canceled his appointment without informing him”. During the scenario, the characters’, i.e., doctor and the patient, their personalities, inner conflicts and dilemmas between them were portrayed as closely to clinical reality, medical systems, laws and regulations. During this enactment, students tried to empathize with the feelings of the patient and understand the thoughts and behaviors. The “doctor” tried to solve the present difficulties by using clinical communication skills learned from the course.

### Feedback and comments

After the enactment, the facilitator asked the actors to step out of their roles and share what they learned from the scenario. This was followed by a period of feedback and discussion whereby the facilitator asked the “doctor” to describe the skills he or she used to resolve the conflict. The observers were also asked to provide feedback. Finally, the “patient” was asked to discuss how he or she felt and to comment on the effectiveness of the “doctor’s” communication skills. The feedback and group discussion sessions were focused particularly on the aspects of communication and lasted approximately 30 min. Afterwards the facilitator summarized the content or focused on issues not identified by the audience to provoke further thought and discussions.

### Assessments

In 2003, the Chinese Medical Doctors Association, in conjunction with relevant professional organizations and drawing on international standards, organized and developed the first standardized training framework and evaluation questionnaire for “Practicing Skills of Chinese physicians in humanistic Medicine” suitable for domestic physicians [27, 28]. On this basis, we incorporated clinical scenario drama as its teaching method, and made appropriate modifications to the curriculum framework and evaluation issues in the actual teaching process, gradually forming the existing self-rating questionnaire.

The self-rating questionnaire used a 5-point Likert scale (1 = strongly disagree, 2 = disagree, 3 = neither agree nor disagree, 4 = agree, 5 = strongly agree) that was designed based on the aims of the course and included 7 items on self-confidence regarding communication skills and professional practice (before and at the end of the course), 8 items regarding self-perceived learning attitude and ability (before and at the end of the course). and 5 items rated on self-perceived satisfied and utility of the course (at the end of the course).

### Statistical analysis

Statistical analyses were performed using IBM SPSS 20.0 and self-contrast methodology. The means and standard deviations were used to describe the total scores and single scores for each item on the self-rating questionnaire. Pre- and post-course scores were compared using paired t test. *p*-value of less than 0.05 (2tailed) were considered statistically significant.

## Results

From March 2009 to October 2018, 727 medical students (M: F 387:340) completed the doctor-patient relationship and clinical communication skills course at PUMC. A total of 727 self-rating questionnaires were issued before the course, with 697 responses collected; 727 were issued at the end of the course, with 675 responses collected. A total of 651 students completed the questionnaire before and at the end of the course. With respect to curriculum evaluation, 727 questionnaires were distributed, with 675 responses received. Despite several verbal reminders, some of the students did not complete the survey (Table. 3).

**Table 3:** Self-rating questionnaires that were issued and collected spanning 10-years of course management

Year	Issued	Collected (before course initiation)	Collected (end of the course)	Both (before & after)
2009	96	88	84	79
2010	74	67	63	60
2011	59	54	52	50
2012	56	52	50	47
2013	65	61	60	57
2014	84	81	84	81
2015	86	82	75	72
2016	71	70	71	70
2017	64	63	64	63
2018	72	72	72	72
Total	727	690	675	651

### Implementation process of the course

At the beginning of the course, many students thought that aim of doctor-patient communication was merely to reduce conflicts between doctors and patients. Our true aim is to help medical students understand the “human relationship” between doctor and patient, as Michael Balint famously said. Reducing conflict was just a natural part of that process.

Students are encouraged to understand the principles, basis and essence of doctor-patient relationship and communication after they finish their studies, and then apply these principles in medical practice. Clinical scenario drama is used as the main teaching method. In the beginning, instructional topics are as covered by the Calgary-Cambridge Guide, which include establishing a trusting doctor-patient relationship, collecting medical history information, explaining medical problems, breaking bad news, and negotiating treatment plan. Also, in accordance with situations in China, we added communication task with patient’s family members. In total, seven themes emerged, including: Relationship building and empathy, obtaining patient psychosomatic history, explaining medical problems, negotiating treatment options, breaking bad news, coping with demanding patients, and communicating with relatives. In addition, case scripts were designed to make it in line with the Chinese context and cultural background (Table 1). During the years, students reported that they preferred clinical scenario dramas to lectures and thought they could gain more from practice. So, we adjusted the didactics with reduced lecture time (about 30 min per class). And a standardized operating procedure of role coaching was designed to guide all teachers (Table 2).

### Results of the self-rating questionnaires and curriculum evaluation

The majority of the study participants had significant improvements (*p*<0.05) in self-confidence regarding communication skills and professional practice, including relationship building, explaining medical problems, negotiating treatment options, breaking bad news, coping with demanding patients, communicating with relatives when compared to pre-course evaluations and measurements (Table 4). Ratings for each subject before and after the course were analyzed using paired t-test, and the results were significantly different. The student curriculum evaluation results suggested that clinical scenario dramas were accepted and recognized for teaching doctor-patient communication skills compared to lectures (Table 5).

**Table 4:** Differences between pre and post-course self-assessments (*N* = 651)

	Mean ± SD	95% CI	P
**I have confidence in the following skills**			
Relationship building	−.313 **±** .917	−.384 ~ −.243	0.000
Psychosomatic history taking	.035 **±** .856	−.031 ~ −.101	0.293
Explaining medical problems	−.442 **±** .914	−.513 ~ −.372	0.000
Negotiating treatment options	−.638 **±** 1.06	−.720 ~ −.557	0.000
Breaking bad news	−.374 **±** .890	−.442 ~ −.305	0.000
Coping with demanding patients	−.396 **±** .894	−.465 ~ −.327	0.000
Communicating with relatives	−.495 **±** .939	−.568 ~ −.423	0.000
**I have changed my learning attitude and ability in the following aspects**			
Take an active part in the learning process	−.848 **±** .853	−.914 ~ −.783	0.000
Apply what I have learned to practice	−.619 **±** .831	−.683 ~ −.555	0.000
Take the initiative to ask for feedback during the learning process	−.711 **±** .926	−.783 ~ −.640	0.000
Ability to learn independently	−.398 **±** .907	−.468 ~ −.328	0.000
Self-discovery and cognition of learning needs	−.594 **±** .866	−.661 ~ −.528	0.000
Ability to learn in a team	−.748 **±** .876	−.815 ~ −.681	0.000
Ability to learn from problem solving	−.429 **±** .914	−.815 ~ −.681	0.000
Ability to learn from past practice experience	−.450 **±** .816	−.513 ~ −.388	0.000

**Table 5:** Overall Curriculum Evaluation

	Mean ± SD (***N*** = 675)
**How helpful were the following activities? Scale from 1 = not helpful to 5 = very helpful**	
Lectures	4.07 **±** 0.831
Clinical scenario drama	4.39 **±** 0.610
P	0.000
**How strongly would you agree or not agree with the following statements? Scale from 1 = do not agree at all to 5 = strongly agree**	
I am satisfied with the whole course	4.61 **±** 0.546
I am satisfied with the facilitators	4.65 **±** 0.535
I am satisfied with the content of the teaching unit	4.63 **±** 0.534

## Discussion

We conducted courses on doctor-patient relationship and clinical communication skills for over 10 years at PUMC using clinical scenario drama techniques. Feedbacks from PUMC medical students indicated that subjectively, clinical scenario drama helped them to gain stronger confidence and better understand the doctor-patient relationship and improve their clinical communication skills, except for psychosomatic history taking.

Few studies have explored the impact of doctors’ low self-confidence in their ability to communicate. Self-confidence affects cognition, emotion, intention and behavior in a wide range of ways [29]. When faced with stress and difficulties, people instinctively choose to use their more confident skills to cope. We have observed in clinical scenario drama that “doctors” are often more willing to use their medical knowledge and skills to persuade patients when confronted with contradictions and conflicts. The reasons why clinical scenario drama can improve the confidence of medical students in some tasks in doctor-patient communication may lie in: (1) Similar to social or psychodramas [30, 31], clinical scenario dramas places medical students in situations felt by patients and their relatives. During these enactments, medical students become aware of the frustrations and challenges felt by their patients. The study participants played the role of patients, family members, or medical staff, so as to experience their attitudes, feelings, and emotions. In addition, the observer group had the opportunity to experience empathy and the highly emotional state of the patients. With regards to Ms. Liu’s clinical scenario drama (Additional file 1), observers were exposed to the feelings behind the roles, the fear, the loneliness, and the helplessness. Uncovering these feelings and allowing them to be expressed is the goal of conducting these clinical scenario dramas. It is a powerful technique to help one “face one’s demons” and is a key aspect for uncovering the unspoken thoughts and feelings that makes clinical communication challenging. In this regard, clinical scenario drama techniques are more effective compared to lectures as it helps improve empathy in medical staff. (2) As is known to all, the doctor-patient communication is a kind of procedural memory, need to be established in constant practice and mature. This kind of practical exercise can undoubtedly promote the confidence of the medical students in the use of various communication skills. Moreover, after receiving the feedback from observers, medical students still have the opportunity to try or observe the practice of other students, which provides the possibility of adopting different communication strategies in the same scene.

In this study, the students did not experience any improvement in their self-perception ability or confidence in taking psychosomatic medical histories. We found no previous studies that evaluated the benefits of drama techniques on psychosomatic history taking. However in 2017 [32], a German cohort study that was based on video-taped consultations gathered from year-four and graduating medical students indicated, on average, final-year students scored higher in differential-diagnostic questioning and in-time management compared to their year-four counterparts. However, these final-year students showed deficiencies in basic communication skills and in interpreting and understanding patients’ concerns, patient’s perspectives and lacked empathy. In this study, there was no significant increase in medical students’ self-confidence in taking psychosomatic history. The reason may be that the students have had previous training in medical history taking and have a certain level of confidence in this area. Through the study of doctor-patient relationship and communication, students realize that they still have shortcomings in this field.

The results of this study also showed an improvement in their self-evaluations for active learning ability. Students felt that they could participate more actively, find solutions to problems, learn better in a team environment, gain insight from feedback and previous experiences. In addition, medical students were more interested in clinical scenario dramas compared to taking lectures. The students were highly satisfied with the course setting, teaching instructors and teaching content.

In reviewing the literature, we did not find any research on the perception and evaluation of drama technology among Chinese mainland medical students. Traditional Oriental culture and education mode are different from that of the West. In the field of medicine, they are more inclined to traditional family decision-making and pay more attention to the authority of doctors in doctor-patient relationship [33]. In the process of medical education, students are usually just recipients and teachers tell students what is right or wrong. The teaching principles of doctor-patient communication introduced from the West pay more attention to patient autonomy, emphasize patient-centered, and students’ participation, experience, feedback and practice in the teaching process. It is doubtful whether such a concept and method can be accepted by medical students in mainland China. Our students’ experience of clinical scenario drama was positive, according to the study. In such learning, medical students are not only “doctors”, but also “patients”, or “patients’ families”; Not just the student, but the teacher, or the provider of feedback. In such a learning process, students not only acquire knowledge, but also experience and skills. As one student responded, “For learning medicine, the things out of medicine make a difference.”

There were several limitations to the current study. First of all, doctor-patient communication is a very complex process, and the existing evaluation methods in this study are not enough to objectively evaluate the impact of courses on students’ actual communication ability. Secondly, there was a lack of control group in the study. Hence it is necessary to further evaluate the relationship between self-confidence and objective ability for doctor-patient communication skills of medical students.

## Conclusion

This study introduced in detail the teaching methods for clinical scenario dramas and systematically evaluated these techniques for improving doctor-patient relationship and clinical communication skills of medical students. This is the first time a study of this nature has been conducted in mainland China. Our results showed that the clinical scenario dramas as a teaching method of doctor-patient relationship and the clinical communication skills, subjectively, enhance the medical students the confidence and the understanding of the various clinical communication task and compared with traditional teaching method, can increase the students’ learning interest and initiative. However, its effect on clinical communication, still remains to be further verified.

## Supplementary Information

**Additional file 1 Appendix 1.** Examples of Clinical scenario dramas. **Appendix 2.** Comparison of pre - and post-course student evaluations for different grades in the last 10 years. **Appendix 3.** Curriculum evaluation for each grade.

## Data Availability

The dataset supporting the conclusions of this article is included within the article.
