# Supplementary material for: Effectiveness of clinical scenario dramas to teach doctor-patient relationship and communication skills
Source: BMC Med Educ. 2020 Nov 26;20:473. doi: 10.1186/s12909-020-02387-9 (PMC7689996; doi:10.1186/s12909-020-02387-9)
Supplement: Supplementary file 1 — Additional file 1 Appendix 1. Examples of Clinical scenario dramas. Appendix 2. Comparison of pre - and post-course student evaluations for different grades in the last 10 years. Appendix 3. Curriculum evaluation for each grade. [file 12909_2020_2387_MOESM1_ESM.docx]

**Appendix 1.** **Examples of Clinical scenario dramas**

| **Theme: CALM model (Coping with demanding patient)**  **Site:** Radiotherapy department ward  **Characters:** Ms. Liu, nurse; Doctor Wang (resident doctor)  Ms. Liu is a 49-year-old patient with advanced nasopharyngeal carcinoma and who is divorced for over 9 years. She was seen by another doctor at an outpatient clinic and is now awaiting admission.  **First act：**  Ms. Liu was notified to complete the admission process. She learned, however, when she arrived at the ward, that there was no room for a female patient on that day. The reason for the mistake was that Doctor Wang, who oversaw patient admissions, mistook her for a man because of her masculine sounding name. Ms. Liu was very disappointed and angry, while Doctor Wang was extremely embarrassed. Dr. Wang had to ask the patient to return to the hotel and wait because there were no rooms available for females. Ms. Liu seemed agitated and remarked, "Doctor, do you know I've been in Beijing for almost three months now? I can wait, but the outpatient doctor told me that I am in advanced stage of cancer. "Dr. Wang apologized again and promised that a female patient would be discharged soon, and Ms. Liu would be hospitalized at that time. With no choice, Ms. Liu left the ward grumbling and went back to the hotel to wait. She said harshly as she left, “Don't make me wait again next time!”  **Second act:**  At 9:00pm on the second night of her hospitalization, Ms. Liu wanted to take a bath and rest, however the husband of her roommate had no intention of leaving. Ms. Liu felt very inconvenienced and requested her roommate’s husband to leave. The husband explained that his wife just had surgery that day and needed someone to take care of her, so he was going to spend the night. Ms. Liu flew into a rage upon hearing this. “The hospital allowed a male relative to stay in a female patient's room at night, but I was not allowed to be hospitalized a week ago just because I was a female patient!” Then, Ms. Liu went to the nurse’s station and shouted at the nurse, “Are the hospital rules only for me?” The nurse was bewildered and asked the doctor on duty to deal with the situation. Coincidentally, the doctor on duty was Dr. Wang. Ms. Liu was even angrier when she saw Dr. Wang and said, “This is my bitter enemy!”  **Third act: (The doctor tries to use the CALM model to pacify the patient)**  Dr. Wang invited the Ms. Liu into his office and asked her to sit down. She listened carefully to the patient, but she appeared very nervous based on her facial expression and body movements. The patient was angry and disappointed with the hospital. “Are all the rules against me?” she yelled. The doctor tried to understand the patient’s emotions and feelings, repeating, “I understand you…I understand you…”. The doctor attempted to explain, saying, “The rules of our hospital are…” , however Ms. Liu became even unhappier, and the conversation ended.  **Feedback and comments**  In the feedback and comments section, we acknowledged the efforts made by the doctor to control the patients’ emotions and the efforts made to understand and empathize with the patient. Observers of the scenario and the person who acted as the patient noted that doctors could do better. For example, facial expressions and body language should convey a more relaxed and confident state of mind. The doctor must recognize the deeper feelings of the patient. For example, in addition to the anger and feelings of being treated unfairly, the patient is fearful of her disease and, as a divorced woman, is likely to have feelings of loneliness and helplessness. The patients’ situation should be understood, and she should be comforted to minimize her anger.  To help study participants master communication skills, we performed a role-play whereby the “doctor” experimented with the above-mentioned ideas and received positive feedback from the “patient”. As a result, the “doctor” displayed greater confidence when communicating with the patient. |
| --- |

**Appendix 2.** Comparison of pre - and post-course student evaluations for different grades in the last 10 years

|  | 2009 | 2010 | 2011 | 2012 | 2013 | 2014 | 2015 | 2016 | 2017 | 2018 |
| --- | --- | --- | --- | --- | --- | --- | --- | --- | --- | --- |
| **I have confidence in the following skills:** |  |  |  |  |  |  |  |  |  |  |
| Relationship building | -.797±.897* | -.283±.885* | -.500±1.11* | -.404±.901* | -.439±.887* | ***.099±.875*** | -.347±.981* | -.243±.859* | ***-.143±.618*** | ***-.167±.856*** |
| Psychosomatic history taking | ***.165±.980*** | ***.080±.829*** | ***.020±.869*** | ***-.106±.598*** | ***.158±.819*** | ***.012±.829*** | ***.111±.912*** | ***.114±.941*** | ***-.016±.813*** | ***-.014±.831*** |
| Explaining medical problems | -.973±.822* | -.617±.922* | -.520±.931* | -.660±.841* | -.754±.786* | -.506±.950* | ***-.167±.856*** | ***-.129±.977*** | ***-.143±.737*** | -.222±.791* |
| Negotiating treatment options | -1.04±1.19* | -.600±1.09* | ***-.340±1.22*** | -.787±1.02* | -.842±.922* | -.432±1.16* | -.500±.979* | -.543±.774* | -.635±1.07* | -.653±.966* |
| Breaking bad news | -.532±.918* | -.417±.869* | -.540±.813* | -.574±1.08* | -.719±1.01* | -.337±.841* | ***-.139±.737*** | -.257±.755* | ***-0.095±.928*** | -.278±.843* |
| Coping with demanding patients | -.772±.999* | -.467±.965* | -.440±.884* | -.553±.855* | -.596±.776* | -.210±.684* | -.431±.932* | -.157±.629* | -.286±.991* | ***-.139±.969*** |
| Communicating with relatives | -.949±.904* | -.350±.820* | -.540±.952* | -.702±1.12* | -.702±1.02* | -.488±.746* | -.310±.919* | -.257±.755* | ***-.238±.103*** | -.437±.967* |
| **Evaluation for learning attitude and ability** |  |  |  |  |  |  |  |  |  |  |
| Take an active part in the learning process | -.759±1.04* | -.587±.816* | -1.20±.904* | -.851±.834* | -.877±.758* | -.612±.834* | -.667±.949* | -.958±.664* | -1.33±.691* | -.806±.816* |
| Apply what I have learned to practice | -.519±.857* | -.444±.857* | -.920±.966* | -.723±.852* | -.772±.756* | -.580±.772* | -.389±.815* | -.324±.692* | -.672±.592* | -.681±.917* |
| Take the initiative to ask for feedback during the learning process | -.595±1.16* | -.873±.924* | -.939±.801* | -.830±940* | -.877±.908* | -.704±1.05* | -.690±.729* | -.507±.694* | -.531±.616* | -.732±.940* |
| Ability to learn independently | -.278±.800* | -.397±.943* | -.700±.763* | -.660±1.07* | -.737±.955* | ***-.123±1.10*** | -.958±.911* | ***-.056±.754*** | -.547±.733* | -.403±.850* |
| Self-discovery and cognition of learning needs | -.544±.844* | -.333±.916* | -.480±.909* | -.681±,980* | -.667±.932* | -.481±.963* | -.430±.899* | -.592±.803* | -.766±.771* | -.746±.769* |
| Ability to learn in a team | -.506±.904* | -.540±.800* | -.800±.700* | -.804±1.03* | -.911±.900* | -.469±.923* | -.958±.911* | -.859±.743* | -.703±.728* | -1.03±.888* |
| Ability to learn from problem solving | -.104±.867* | -.429±.928* | -.500±1.06* | -.617±.968* | -.702±.866* | -.395±.958* | -.430±.899* | -.451±.733* | -.484±.873* | -.375±.926* |
| Ability to learn from past practice experience | -.430±.812* | -.317±.877* | -.560±.760* | -.723±,902* | -.772±.824* | -.309±1.02* | -.361±.775* | -1.02±.635* | -.359±.601* | -.481±.874* |

* P < 0.05

**Appendix 3.** Curriculum evaluation for each grade

|  | 2009 | 2010 | 2011 | 2012 | 2013 | 2014 | 2015 | 2016 | 2017 | 2018 |
| --- | --- | --- | --- | --- | --- | --- | --- | --- | --- | --- |
| **How helpful were the following activities? Scale from 1 = not helpful to 5 = very helpful** | | | | | | | | | | |
| Lectures | 3.92±.810 | 3.90±.665 | 3.92±.913 | 3.94±.740 | 3.92±.743 | 3.93±.768 | 4.18±.812 | 4.17±.910 | 4.27±.761 | 3.95±.998 |
| Clinical scenario drama | 4.46±.525 | 4.35±.513 | 4.33±.653 | 4.50±.544 | 4.52±.537 | 4.41±.729 | 4.36±.605 | 4.44±.499 | 4.72±.453 | 4.44±.577 |
| P | 0.000 | 0.000 | 0.015 | 0.000 | 0.000 | 0.000 | 0.102 | 0.051 | 0.000 | 0.001 |
| **How strongly would you agree or not agree with the following statements? Scale from 1 = do not agree at all to 5 = strongly agree** | | | | | | | | | | |
| I am satisfied with the whole course | 4.82±.415 | 4.43±.499 | 4.63±.528 | 4.48±.505 | 4.53±.503 | 4.11±.724 | 4.74±.443 | 4.68±.471 | 4.94±.244 | 4.75±.434 |
| I am satisfied with the facilitators | 4.85±.396 | 4.48±.564 | 4.61±.568 | 4.68±.471 | 4.68±.469 | 4.40±.710 | 4.67±.526 | 4.56±.499 | 4.94±.244 | 4.68±.524 |
| I am satisfied with the content of the teaching unit | 4.82±.415 | 4.48±.535 | 4.55±.541 | 4.52±.505 | 4.50±.537 | 4.38±.738 | 4.70±.462 | 4.61±.492 | 4.94±.244 | ***4.70***±.462 |
